# Sleep in Functional Motor Disorders: A Case–Control Polysomnographic Study

**DOI:** 10.1111/jsr.70163

**Published:** 2025-07-31

**Authors:** Jiří Nepožitek, Simona Dostálová, Martin Jirásek, Gabriela Chaloupková, Zuzana Forejtová, Lucia Nováková, Veronika Rottová, Veronika Konvičná, Karel Šonka, Mark J. Edwards, Tereza Serranová

**Affiliations:** ^1^ Department of Neurology and Center of Clinical Neuroscience First Faculty of Medicine, Charles University and General University Hospital Prague Czech Republic; ^2^ Department of Rehabilitation and Sports Medicine Second Faculty of Medicine, Charles University and University Hospital Motol Prague Czech Republic; ^3^ Institute of Psychiatry, Psychology and Neuroscience, King's College London London UK

**Keywords:** functional motor disorder, functional movement disorder, functional neurological disorder, movement disorders, polysomnography, REM sleep

## Abstract

Sleep problems are frequent in functional motor disorders (FMDs). Surprisingly, objective correlates of impaired sleep and its relationship to other comorbidities have been understudied, and no polysomnographic study is available. We aimed to map the polysomnographic parameters in the context of self‐reported sleep and mood symptoms and search for comorbid sleep disorders in FMD and healthy controls. Thirty‐seven patients (mean age [SD], 48.2 [10.6] years) with clinically definite FMD and 37 controls (48.6 [11.2] years) underwent structured medical and sleep history assessment, neurological examination and polysomnography and completed questionnaires for sleep quality, sleepiness, depression and anxiety. In FMD, specific sleep disorders were identified in our cohort, with 32% having restless legs syndrome, 38% clinically significant obstructive sleep apnoea and 8% periodic limb movements in sleep. FMD patients reported worse sleep quality (*p* < 0.001), higher sleepiness (*p* < 0.001), depression (*p* < 0.001) and anxiety (*p* < 0.001), had longer REM sleep latency (*p* < 0.001), worse sleep efficiency (*p* = 0.012) and increased wake ratio (*p* = 0.013). Furthermore, longer sleep latency (*p* = 0.030) and decreased REM sleep ratio (*p* = 0.027) in FMD reached nominal significance before adjustment for multiple comparisons. In FMD, subjective sleep quality positively correlated with depression (*ρ* = 0.54; *p* < 0.002) and anxiety (*ρ* = 0.61; *p* < 0.001) and subjective sleepiness correlated with depression (*ρ* = 0.42; *p* = 0.010). Self‐reported measures did not correlate with any polysomnographic parameters. Polysomnography detected sleep structure changes in FMD. Sleep abnormalities, including impairments in REM sleep, should be considered in the management of FMD. Future studies should further explore the role of REM sleep disturbances in the pathophysiology of FMD.

## 1 Introduction

Functional motor disorder (FMD) is a common condition and an important source of disability in neurology (American Psychiatric Association 2013). The clinical presentation of FMD is typically variable and complex, with numerous motor and non‐motor symptoms coexisting in one individual. Non‐motor symptoms, including pain, fatigue, mood disorders and cognitive complaints, have consistently been identified as the major determinants of health‐related quality of life (Butler et al. 2021; Forejtova et al. 2023; Gelauff et al. 2020; Nicholson et al. 2020; Vechetova et al. 2018). Sleep problems, including insomnia, low efficiency, short sleep and increased sleepiness, are reported by the majority of patients with FMD and represent the fourth most frequent non‐motor symptom after fatigue, pain and sensory symptoms (Carson et al. 2015; Graham and Kyle 2017; Stone et al. 2010; Vechetova et al. 2018). In FMD patients, comorbid non‐motor symptoms are often considered to be of functional aetiology; however, they could also result from other neurological disorders, including unrecognised sleep disorders such as obstructive sleep apnoea (OSA), restless legs syndrome (RLS) and periodic limb movements of sleep (PLMS). Furthermore, fatigue, pain, mood disorders and sleep disorders influence each other (Corfield et al. 2016; Finan et al. 2013; Hell 2015; Lopez et al. 2017; Pandi‐Perumal et al. 2020; Rakel 1999; Yasugaki et al. 2025), and understanding the complex inter‐relationship is important for tailoring treatments in FMD (Lim et al. 2020).

In FMD, objective correlates of impaired sleep, its relationship to other comorbidities and its impact on health‐related quality of life have been understudied. Larger epidemiological studies mapping the frequency of sleep comorbidities in FMD are lacking, and only a few studies used objective sleep assessment to provide a deeper understanding of sleep quality and associated phenomena. Our group has recently utilised polysomnography (PSG) and multiple sleep latency test (MSLT) parameters to investigate the reported increase in sleepiness among individuals with FMD and central hypersomnia (Nepozitek et al. 2024). While we did not find objective correlates of increased sleepiness in FMD patients in contrast to those with central hypersomnia, we observed a higher frequency of sleep comorbidities, such as OSA and RLS. Previously, we documented an increased prevalence of RLS and clinically significant PLMS assessed actigraphically in a relatively large cohort of FMD patients (Serranova et al. 2019).

PSG is a key diagnostic tool in sleep medicine and is also used to demonstrate the pathophysiological mechanisms underlying currently known sleep disorders. It is essential to determine both sleep quantity and quality by scoring sleep stages, which are equally crucial for sleep health (McCarter et al. 2022). Abnormalities in PSG parameters across various sleep stages have been associated with impairments in cognitive and emotional processes and linked to various neuropsychiatric disorders (Krystal 2020). A PSG study comparing sleep data in individuals with FMD to a cohort of healthy controls has not yet been performed.

The current study aimed to map the PSG parameters in the context of self‐reported sleep quality, sleepiness, depression and anxiety, to search for comorbid sleep disorders in individuals with FMD, and to compare them with healthy controls. We hypothesised that objective parameters of disturbed night sleep, together with self‐evaluation of sleep quality, sleepiness, depression and anxiety, would be increased in people with FMD.

## 2 Methods

### 2.1 Study Participants

Thirty‐seven patients with clinically definite FMD (mean age 48.2 [standard deviation (SD) 10.6] years); mean disease duration 4.2 (SD 2.8) years; mean Simplified Functional Movement Disorders Rating Scale (S‐FMDRS) (Nielsen et al. 2017) 14.6 (SD 7.8) (range: 2–26) who reported any sleep difficulty and agreed with the sleep examination were included in the study. The diagnosis of FMD was based on the presence of positive signs of internal inconsistency in motor symptoms during one task or between different tasks. All patients fulfilled the Gupta and Lang diagnostic criteria for clinically definite FMD (Gupta and Lang 2009), and also fulfilled the criteria for motor functional neurological symptom (conversion) disorder according to *The Diagnostic and Statistical Manual of Mental Disorders*, Fifth Edition (DSM‐5) (American Psychiatric Association 2013). Patients were recruited from the specialised service for FMD patients at the Department of Neurology and Center of Clinical Neuroscience, First Faculty of Medicine, Charles University and General University Hospital in Prague. The exclusion criteria included age under 18, the presence of significant medical illness or any acute health disturbance, a history of major depression, substance dependence or psychosis and a history of an organic neurological disorder of the brain. The study was approved by the local ethics committee, and all participants gave their written consent to participate.

The control cohort consisted of 37 subjects (mean age 48.6 [SD 11.2] years) recruited from the general community through advertisements and matched for age and gender. To be eligible for the study, controls had to be free of major neurologic disorders, active oncologic illness and abuse of psychoactive substances. The exclusion criterion was any neurological disease and current depressive mood. None of the control subjects used any drugs potentially affecting sleep or other centrally acting drugs.

The chronic use of drugs that potentially affect sleep or other centrally acting drugs was recorded in all FMD subjects (Table S1). To distinguish the effect of particular antidepressant groups, the use of ‘REM‐reducing drugs’ and ‘hypnotic REM‐indifferent drugs’ was documented according to their common effect on sleep. The ‘REM‐reducing drug’ category contained drugs prolonging REM sleep latency and suppressing the amount of REM sleep, which is typical for selective serotonin reuptake inhibitors (SSRIs), serotonin and norepinephrine reuptake inhibitors (SNRIs), tricyclic antidepressants (TCA) and pregabalin. The ‘hypnotic REM‐indifferent drug’ category contained drugs reducing sleep latency, increasing sleep efficiency and having inconclusive effects on REM sleep amount and latency, which is typical for mirtazapine and trazodone (Abad and Guilleminault 2005). All subjects were instructed to refrain from smoking and drinking either caffeinated beverages or alcohol on the day of the study. Control participants were not using any hypnotics or other sedatives at the time of PSG recording.

### 2.2 Examination Protocol

The study participants underwent a standard protocol consisting of a comprehensive medical and sleep history, neurological examination, questionnaires as detailed below and nocturnal PSG. None of the participants had significant structural lesions of the brain on the magnetic resonance imaging scan. In the face‐to‐face interview, all subjects were systematically asked about their sleep history (sleep regime, circumstances of falling asleep and waking up, sleep continuity, symptoms of insomnia, symptoms of RLS, OSA, parasomnias, sufficiency of sleep and daytime wakefulness/sleepiness).

To quantify the subjective level of sleep quality, the Pittsburgh Sleep Quality Index (PSQI) was obtained from the study participants (0–45 points) (Buysse et al. 1989). All subjects assessed subjective levels of daytime sleepiness using the ESS (0–24 points) (Johns 1991). To quantify the level of depression, the Beck Depression Inventory, second edition (BDI‐II), was used (0–39 points) (Beck et al. 1961). Anxiety was assessed by the State‐Trait Anxiety Inventory (STAI X2) (Spielberger et al. 1970).

### 2.3 Sleep Examination

The nocturnal PSG was performed using a digital PSG system (RemLogic, version 3.4.1, Embla Systems) and consisted of electrooculography (EOG: E1‐M2, E2‐M2), electroencephalography (EEG: F3‐M2, C3‐M2, O1‐M2, F4‐M1, C4‐M1, O2‐M1), the surface electromyography (EMG) of the bilateral mentalis muscle and the bilateral tibialis anterior muscle, electrocardiography, nasal pressure, nasal and oral airflow, thoracic and abdominal respiratory effort, oxygen saturation and microphone and digitally synchronised video monitoring, measured during the period from 10 PM to 6 AM according to the American Academy of Sleep Medicine (AASM) recommendation (Berry et al. 2015). All features on PSG were analysed visually. The sleep stages, arousals, respiratory events and limb movements were scored according to the AASM Manual for the Scoring of Sleep and Associated Events version 2.22015 (Berry et al. 2015).

The Apnoea–Hypopnoea Index (AHI) was used to define the severity of OSA. The AHI was calculated as the number of respiratory events per hour of sleep. Mild OSA was based on AHI ≥ 5 and < 15, moderate OSA on AHI ≥ 15 and < 30 and severe OSA on AHI ≥ 30 (AASM 2014). To define the hypoxic burden of subjects in sleep, the oxygen desaturation index (ODI) and average saturation during sleep were calculated. ODI was calculated as the average number of desaturation episodes occurring per hour, where desaturation episodes were defined as a decrease in the mean oxygen saturation of ≥ 3% (over the last 120 s) that lasted for at least 10 s. Patients with AHI ≥ 15 were considered to have a clinically significant OSA, indicating the need for continuous positive airway pressure (CPAP) therapy.

The PLMS was based on the AASM criteria (ICSD‐3). The periodic limb movements index (PLMI) quantified the frequency of periodic limb movements per hour of sleep. According to the ICSD‐3, a PLMI > 15 was considered abnormal (AASM 2014).

RLS was diagnosed clinically according to the International RLS Study Group criteria (Allen et al. 2014).

A complaint of insomnia was recorded when subjective difficulty initiating sleep, maintenance of sleep or final awakening earlier than desired was reported during the structured interview, associated with concern, dissatisfaction or perceived daytime impairment (AASM 2023).

### 2.4 Statistics

Categorical variables were analysed using Fisher's exact test. The Mann–Whitney *U* test was used to test group differences. The statistical significance level was set at *p* < 0.05. The Benjamini–Hochberg false discovery rate was applied to adjust for multiple testing. The Spearman correlation coefficient was used for the correlation analysis. ANCOVAs were applied to adjust for the effect of selected relevant parameters. The statistical analysis was performed in SPSS Software (IBM SPSS Statistics Version 26).

## 3 Results

### 3.1 Comparison of Quantitative Results

Patients with FMD had higher scores in PSQI, ESS, BDI‐II, STAI X2, lower sleep efficiency, longer REM sleep latency and increased wake ratio. Although we observed nominally significant longer sleep latency and decreased REM sleep ratio in FMD, these results were no longer tenable with the chosen adjustment for multiple comparisons. Overall comparison analysis results are presented in Table 1.

**TABLE 1:** Demographic, clinical and polysomnographic parameters.

	FMD		Controls		*p*	*p* adj
	Mean	SD	Mean	SD		
Age	48.2	10.6	48.6	11.2	0.850	0.895
Gender (F/M)	27/10		27/10		1.000	1.000
BMI	27.3	4.3	26.5	4.0	0.421	0.526
Questionnaires						
PSQI	25.1	6.7	11.2	3.6	**< 0.001**	**0.004** ^a^
ESS	10.4	5.6	5.9	4.2	**< 0.001**	**0.004** ^a^
BDI‐II	18.4	11.5	3.1	3.3	**< 0.001**	**0.004** ^a^
STAI X2	49.4	9.5	33.6	7.3	**< 0.001**	**0.004** ^a^
Polysomnography						
Total sleep time (min)	340.3	84.6	373.9	77.8	0.121	0.220
Sleep latency (min)	26.3	27.0	16.3	13.0	**0.030**	0.067
Sleep efficiency (%)	70.5	16.8	79.7	11.9	**0.012**	**0.037** ^a^
Sleep stage R latency (min)	164.2	85.6	101.8	61.0	**< 0.001**	**0.004** ^a^
AHI	15.0	16.8	10.9	12.5	0.496	0.562
ODI	9.5	11.5	6.8	8.8	0.408	0.526
Wake (%)	26.0	16.2	17.4	11.4	**0.013**	**0.037** ^a^
Sleep stage N1 (%)	6.7	3.3	7.2	3.1	0.282	0.403
Sleep stage N2 (%)	33.9	9.9	37.9	8.7	0.144	0.240
Sleep stage N3 (%)	19.8	9.8	20.6	8.4	0.506	0.562
Sleep stage R (%)	13.7	7.0	16.9	4.7	**0.027**	0.067
PLMI	3.4	6.8	1.5	4.3	0.158	0.243
Arousal index	22.8	11.8	18.3	12.1	0.109	0.218

*Note*: Data presented as mean and standard deviation, except for gender, where absolute counts are provided. *p* < 0.05 are bolded.

Abbreviations: adj, adjusted; AHI, Apnoea–Hypopnoea Index; BDI‐II, Beck Depression Inventory, Second Edition; BMI, body mass index; ESS, Epworth Sleepiness Scale; F, females; FMD, functional motor disorder; M, males; N1, 2, 3, non‐rapid eye movement sleep stage 1, 2, 3; ODI, oxygen desaturation index; PLMI, periodic limb movements index; PSQI, Pittsburgh Sleep Quality Index; R, rapid eye movement sleep; S‐FMDRS, Simplified Functional Movement Disorders Rating Scale; SD, standard deviation; STAI X2, State‐Trait Anxiety Inventory.

^a^ Significant after Benjamini–Hochberg false discovery rate adjustment.

### 3.2 Sleep Findings and Categorical Comparison

There was no significant difference in the prevalence of OSA, which was found in more than half of the FMD patients, and over 45% of controls had OSA. In FMD, clinically significant OSA prevailed over mild, while in the control group, mild OSA was more frequent than higher‐severity forms; however, the difference was not significant. RLS and complaints of insomnia were significantly more prevalent in FMD. There was no between‐group difference in PLMS frequency. Table 2 presents detailed results of the sleep findings, their prevalence and comparison.

**TABLE 2:** Prevalence of sleep findings and qualitative comparison with controls.

	FMD		Controls		*p*	*p* adj
	*N*	%	*N*	%		
OSA	20	54.1	17	45.9	0.642	0.769
Clinically significant OSA	14	37.8	8	21.6	0.203	0.338
RLS	12	32.4	0	0.0	**0.001**	**0.005** ^a^
PLMS	3	8.1	1	2.7	0.615	0.769
Insomnia	21	56.8	0	0.0	**< 0.001**	**0.005** ^a^

*Note*: Data presented as absolute counts, percentages and *p* values obtained with two‐tailed Fisher's exact. *p* < 0.05 are bolded.

Abbreviations: adj, adjusted; CPAP, continuous positive airway pressure; OSA, obstructive sleep apnoea; PLMS, periodic limb movements in sleep; RLS, restless legs syndrome.

^a^ Significant after Benjamini–Hochberg false discovery rate adjustment.

To assess the relationships of the obtained parameters to insomnia complaints in FMD, we performed a comparison between the groups with and without reported insomnia. The only difference found in an exploratory phase was related to the frequency of RLS, which was no longer tenable with the correction for multiple comparisons. The comparison analysis of insomnia is presented in Table S2.

### 3.3 Effect of Relevant Parameters

To adjust for the effect of depression, anxiety and antidepressant use, ANCOVAs with BDI‐II, STAI X2, REM‐reducing drugs use and hypnotic REM‐indifferent drugs use as covariates were performed. When adjusted for BDI‐II, differences in sleep latency, sleep efficiency, sleep stage R latency, wake ratio and sleep stage R ratio remained significant. When adjusted for STAI X2, ANCOVA analyses failed to show a significant difference in sleep efficiency and wake ratio. In contrast, differences in sleep latency, sleep stage R latency and sleep stage R ratio remained significant. When adjusted for REM‐reducing drugs, differences in PSG parameters, including sleep stage R latency and sleep stage R ratio, remained significant. When adjusted for hypnotic REM‐indifferent drugs, differences in sleep latency, sleep efficiency, sleep stage R latency and wake ratio remained significant. The complete results of the ANCOVA analyses can be seen in Table S3.

We conducted an ANCOVA including all four covariates (BDI‐II, STAI‐X2, REM‐reducing drugs and REM‐indifferent drugs) in a single model to examine their combined influence on group differences. In this model, the group difference remained significant for sleep latency, sleep efficiency, sleep stage R latency and sleep stage R ratio, while the effect for wake ratio was no longer significant. The complete results of the ANCOVA analysis can be seen in Table S4.

### 3.4 Correlation Analysis

In FMD patients, we assessed the correlation of subjective measures of sleep symptoms with self‐reported severity of non‐motor symptoms, use of antidepressants, objectively rated motor severity (S‐FMDRS) and PSG measures. PSQI was positively correlated with BDI‐II (*ρ* = 0.54; *p* < 0.002) and STAI X2 (*ρ* = 0.61; *p* < 0.001). Also, the positive correlation of PSQI to the use of REM‐reducing antidepressants was found, although only borderline (*ρ* = 0.37; *p* = 0.043). ESS was positively correlated with BDI‐II (*ρ* = 0.42; *p* = 0.010). BDI‐II and STAI X2 were positively correlated with REM‐reducing antidepressant use (*ρ* = 0.57; *p* < 0.001; resp. *ρ* = 0.56; *p* < 0.001). PSQI and ESS did not correlate with any objectively measured PSG parameters. Disease duration and severity (S‐FMDRS) did not correlate with other observed parameters.

## 4 Discussion

This study is the first to investigate the PSG profile and self‐reported severity of sleep‐relevant symptoms in FMD compared to healthy controls. In the FMD group, we found increased REM sleep latency, decreased sleep efficiency and increased wake ratio during night sleep, along with subjective reports of impaired sleep quality, increased daytime sleepiness, depression and anxiety. In addition, prolonged sleep latency and decreased REM sleep stage ratio in the FMD group reached nominal significance before adjustment for multiple comparisons. Self‐reported sleep quality impairment in FMD correlated with levels of depression, anxiety and antidepressant use but not with objectively measured PSG parameters.

### 4.1 PSG

Prolonged REM sleep latency was the most robust PSG abnormality in the FMD group. The differences found in REM sleep latency and increased sleep latency (nominally significant before adjustment for multiple comparisons) remained unchanged after adjustment for the effect of psychological confounders such as anxiety, depression and drug effects, suggesting that these PSG measures could represent a sleep signature of FMD.

Increased levels of depression found in FMD are compatible with current knowledge that depression is a frequent comorbidity in FMD (Nicholson et al. 2020; Vechetova et al. 2018). Patients with depression have previously been reported to exhibit specific PSG abnormalities in sleep, such as shortened REM sleep latency, an increase in REM density, as well as total REM sleep time and a decrease in electroencephalogram delta power during non‐REM sleep (Riemann et al. 2020; Yasugaki et al. 2025). These effects with regard to REM sleep latency are opposite to those found in our FMD cohort, despite their increased levels of depression, indicating that they are of potential significance as a more specific feature of FMD.

The differences in REM sleep latency were also independent of medication use. While common antidepressants, such as SSRIs, SNRIs, TCAs and pregabalin, are known to prolong REM sleep latency and reduce REM sleep amount, others, like mirtazapine and trazodone, show inconclusive effects (Abad and Guilleminault 2005). We accounted for these effects by categorising medications based on their effects on sleep for the ANCOVA and correlation analyses, and we found that REM‐reducing drugs did not influence our PSG results.

Our observations of reduced REM sleep, including prolonged REM sleep latency and decreased proportion of REM sleep during the night, suggest that the physiological functions of REM sleep may be impaired in FMD. While the exact role of REM sleep in normal human physiology remains incompletely understood, it is potentially associated with critical functions such as forming and consolidating certain types of memory and motor learning (Peever and Fuller 2017). Cognitive symptoms are very common in people with FMD and other functional neurological or somatic symptom disorders, and have a major impact on their health‐related quality of life (Forejtova et al. 2023; Teodoro et al. 2018; Vechetova et al. 2018). REM sleep impairment may be directly linked to the pathophysiology of cognitive symptoms in FMD or act as a contributing and perpetuating factor. However, there are a number of remaining questions. Whether sleep disturbances are merely incidental in FMD, or represent a useful biomarker, remains unclear. Similarly, it is not clear what the sleep PSG profiles of other subtypes of functional neurological disorder (FND), including functional cognitive disorder, are. Self‐reported sleep disturbance is highly prevalent in functional cognitive disorder and serves as a distinguishing diagnostic feature from neurodegenerative causes; however, there is a lack of PSG studies in this area (Cabreira et al. 2023). Further studies should investigate the relationship between sleep parameters and objective measures obtained from neuropsychological and psychometric/psychophysical assessments (e.g., attentional measures, reaction times) to enhance our understanding of the mechanisms underlying FND, including functional cognitive symptoms.

Emerging evidence suggests that REM sleep may help induce wakefulness by stimulating the central nervous system (Brooks and Peever 2016). Sleepiness, which was also increasingly reported in FMD patients compared to controls, is a frequent self‐reported symptom in FMD (Nepozitek et al. 2024; Vechetova et al. 2018). Although no objective correlation or cause was found in sleep tests (MSLT and PSG) (Nepozitek et al. 2024), sleepiness may reflect an insufficient readiness for wakefulness normally induced by sufficient REM sleep (Brooks and Peever 2016).

REM sleep is also essential for emotion regulation (Kimura et al. 2014). In FND, including FMD, there is evidence pointing to abnormal interoception and emotion processing and regulation (Hallett et al. 2022; Pick et al. 2019; Sojka et al. 2018). Abnormalities in the stress pathway, including abnormal cortisol levels, baseline arousal, and autonomous system abnormalities (e.g., heart rate variability) (Sundararajan et al. 2016), can also interplay with sleep (Hallett et al. 2022). Loss of REM sleep, thus, may contribute to impaired regulation of emotional control in FMD.

The PSG findings from our study are similar to most of the findings from fibromyalgia patients (Wu et al. 2017), particularly sleep latency, sleep efficiency, REM latency, wake ratio and sleep stage REM ratio. The fact that we did not find differences in the other PSG parameters that were found to be different in the meta‐analysis could be due to the greater night‐to‐night variability of these parameters when evaluated on a smaller sample. Another explanation is that these differences are not as pronounced in FMD. The finding of prolonged REM sleep latency in FMD is in contrast with findings from a single PSG study in individuals with functional/dissociative seizures, which is another important subtype of FND. This study showed an abnormally high proportion of REM sleep compared to other sleep stages in a small group of patients with functional seizures, that is, a pattern corresponding to depression (Bazil et al. 2003).

Although the alteration in REM latency observed in our FMD cohort may suggest a specific neurophysiological pattern, direct comparisons to other FND subtypes are currently constrained by the paucity and heterogeneity of available data (Bazil et al. 2003; Kannan et al. 2025; Latreille et al. 2018; Popkirov et al. 2019; Vanek et al. 2021). Existing PSG studies on functional seizures or functional cognitive disorder typically involve small, clinically diverse samples and report inconsistent findings with regard to REM parameters. In some cases, contradictory results, such as both prolonged and shortened REM latency, have been described (Vanek et al. 2021). Furthermore, a substantial proportion of the literature relies solely on subjective sleep complaints without PSG confirmation, thereby limiting physiological interpretation (Latreille et al. 2018). This lack of consistency and heterogeneity of reported measures precludes firm conclusions regarding transdiagnostic sleep architecture patterns within FND. Nonetheless, the observed prolongation of REM latency in FMD contrasts with the REM shortening typically seen in depression, which may lend preliminary support to the specificity of our findings.

The differences in sleep efficiency and wake ratio during sleep were no longer significant after adjustment for the effect of anxiety scores. Given that multiple sleep disturbances, including decreased total sleep time, sleep continuity and sleep depth, are well documented in anxiety‐related disorders (Cox and Olatunji 2020), anxiety likely influenced these parameters in our cohort, and these abnormalities are not specific to FMD.

### 4.2 Comorbidities

Except for RLS and insomnia, the prevalence of sleep disorders did not differ between FMD and controls. This suggests that PSG differences are not driven by particular sleep disorders.

The high frequency of clinically diagnosed RLS (32.4%) is in agreement with previous observations of frequent RLS in FMD (43.8%) (Serranova et al. 2019). However, the prevalence of RLS was not accompanied by proportionally increased prevalence or severity of PLMS. PLMS is a useful but non‐specific biomarker that can support, but not confirm, the diagnosis of RLS. As the diagnosis of RLS is based on clinical criteria, it can be particularly challenging in patients with FMD, where functional mimics may be present. In this study, subjects with inconsistent or atypical reports of RLS symptoms were considered to have functional symptoms and were classified as RLS negative. Moreover, PLMS may be absent or fall below diagnostic thresholds in some individuals with RLS, especially in milder cases. The low PLMS index found in this study contrasts with previous findings of our group (Serranova et al. 2019). In the previous study using three‐night actigraphy, a simultaneous occurrence of RLS and actigraphically established PLMS (PLMI ≥ 22.5/h), supportive of RLS diagnosis, was identified in 21.2% of patients with FMD and only 2.6% of healthy controls. This discrepancy highlights the complexity of distinguishing true RLS from functional mimics in this population.

Although OSA was found to be present at a corresponding frequency in both groups to the general population (Senaratna et al. 2017), clinically relevant OSA indicated for CPAP therapy was present at a slightly higher frequency in FMD than in controls. We have previously reported the comorbidity of OSA in FMD (Nepozitek et al. 2024). In both patients and controls, a similarly increased BMI was found, with, on average, the groups both being overweight, which is a known risk factor for OSA. In the patient group, this increased weight could also be linked to antidepressants and/or immobility.

Although FMD patients reported insomnia complaints at a relatively high frequency (56.8%), we did not find any relations to disease severity, depression and anxiety scores, PSG parameters and sleep comorbidities. Subjective insomnia complaint can be underlined by a phenomenon known as sleep state misperception, where objective measurements reveal a normal quality and duration of sleep (Bastien et al. 2014). In FND, disorders of interoception and incorrect predictive models regarding somatic functions have been demonstrated (Van den Bergh et al. 2017). Therefore, insomnia complaints in patients with FMD could result from sleep state misperception, which is supported by a lack of correlations with objective PSG parameters (Nepozitek et al. 2024). Misperception of sleep may thus be analogous to misperception of movement, sensitivity, pain and other functional symptoms of FND.

### 4.3 Reported Sleepiness and Sleep Quality

In line with previous findings of increased self‐reported poor sleep quality in FND, including FMD, the patients with FMD reported significantly impaired sleep quality compared to controls (Graham and Kyle 2017). In FMD, the level of self‐reported sleep quality impairment was not related to any of the polysomnographically measured parameters. Previously, it was reported that in the general population, sleep quality is closely related to total sleep time, sleep efficiency and slow‐wave sleep duration (Åkerstedt et al. 2019; Kaplan et al. 2017; Keklund and Akerstedt 1997). The absence of correlation between subjective sleep quality and sleepiness, and objective PSG measures in FMD is a key and clinically relevant finding. This mismatch was also observed in our prior study investigating correlates of sleepiness in FMD, in which we found no objective evidence of increased physiological sleep propensity on the MSLT, despite subjective reports of excessive daytime sleepiness comparable to those of individuals with hypersomnia (Nepozitek et al. 2024). Together, these findings reinforce growing evidence that in FMD, subjective symptom experiences frequently do not align with physiological or behavioural measures (Adewusi et al. 2021; Jungilligens et al. 2022; Slovak et al. 2022).

This dissociation is consistent with predictive coding models of symptom generation, which propose that symptoms in FMD and related disorders arise not solely from bottom‐up sensory input, but from abnormal top‐down expectations about bodily states (Barrett et al. 2016; Shaffer et al. 2022; Van den Bergh et al. 2017). Furthermore, the current frameworks for FMD highlight the role of altered interoception and disordered allostatic regulation in shaping the perception and experience of symptoms (Drane et al. 2021; Edwards et al. 2012; Jungilligens and Perez 2024; Van den Bergh et al. 2017). In FMD, this framework is supported by converging evidence of interoceptive disturbances (Demartini et al. 2016; Ricciardi et al. 2016, 2021; Sojka et al. 2021) and a higher prevalence of alexithymia (Demartini et al. 2014). Our current findings of altered perceptions of sleep and sleepiness in FMD without accompanying PSG correlates of common sleep disorders further highlight the potential role of abnormal interoception in multiple aspects of FND, including sleep.

## 5 Limitations

There are limitations to this study that should be noted. First, some of the patients used medication, potentially affecting sleep. The use of multiple medication types that potentially have such adverse effects is a frequent burden on patients with FMD, which is hardly avoidable in clinical studies. In our study, it enabled us to consider the possible effect of antidepressant use.

In line with these findings, we suggest that antidepressant use affecting REM sleep should be carefully considered in FMD. For the treatment of depression, anxiety and insomnia, pharmacological treatment with antidepressants should be part of more complex interventions, for example, therapies based on the cognitive‐behavioural protocol. The relationship between sleep and psychiatric disorders is bidirectional. Poor quality sleep can be stressful for the individual, and the stress leads to the deterioration of mental health and contributes to the development of psychiatric disorders (Wichniak et al. 2017).

Another limitation of the study is that we did not perform a standard insomnia assessment, and quantitative data are not available; insomnia complaints were assessed through a structured interview. Also, a selection bias cannot be ruled out regarding the data on insomnia complaints.

## 6 Conclusion

PSG detects changes in sleep structure in FMD, particularly increased REM sleep latency, sleep latency and wake ratio during night sleep, decreased sleep efficiency and REM sleep stage ratio. Impaired REM sleep should be considered as part of the management of FMD and in future studies regarding the pathophysiology of FMD. Impaired sleep quality reported by patients with FMD seems to be independent of any primary sleep disorder. It rather results from abnormalities in complex higher‐level perceptual and self‐referential processes. The poor correlation between objective and subjective sleep parameters makes formal PSG particularly important to perform in people with FMD.

## Author Contributions

**Jiří Nepožitek:** conceptualization, methodology, data curation, investigation, formal analysis, writing – original draft. **Simona Dostálová:** investigation. **Martin Jirásek:** data curation. **Gabriela Chaloupková:** investigation, data curation. **Zuzana Forejtová:** investigation. **Lucia Nováková:** investigation, data curation. **Veronika Rottová:** data curation, investigation. **Veronika Konvičná:** investigation. **Karel Šonka:** writing – review and editing. **Mark J. Edwards:** writing – review and editing. **Tereza Serranová:** conceptualization, methodology, funding acquisition, data curation, supervision, resources, writing – review and editing, project administration, investigation.

## Ethics Statement

The study was approved by the local ethics committee.

## Consent

All participants gave their written consent to take part in the study.

## Conflicts of Interest

The authors declare no conflicts of interest.

## Supporting information


**Table S1:** Overview of the clinical parameters among the FMD group.
**Table S2:** Comparison of demographic, clinical and polysomnographic parameters in FMD patients with and without insomnia.
**Table S3:** ANCOVA analyses showing a comparison of polysomnographic parameters with BDI‐II, STAI X2 and antidepressant use as covariates.
**Table S4:** ANCOVA with combined influence of covariates—BDI‐II, STAI X2, REM‐reducing and REM‐indifferent antidepressants.

## Data Availability

The data that support the findings of this study are available on request from the corresponding author. The data are not publicly available due to privacy or ethical restrictions.

## References

[jsr70163-bib-0001] Abad, V. C. , and C. Guilleminault . 2005. “Sleep and Psychiatry.” Dialogues in Clinical Neuroscience 7, no. 4: 291–303. 10.31887/DCNS.2005.7.4/vabad. 16416705 PMC3181745

[jsr70163-bib-0002] Adewusi, J. , L. Levita , C. Gray , and M. Reuber . 2021. “Subjective Versus Objective Measures of Distress, Arousal and Symptom Burden in Patients With Functional Seizures and Other Functional Neurological Symptom Disorder Presentations: A Systematic Review.” Epilepsy & Behavior Reports 16: 100502. 10.1016/j.ebr.2021.100502. 34917921 PMC8669370

[jsr70163-bib-0003] Åkerstedt, T. , J. Schwarz , G. Gruber , J. Theorell‐Haglöw , and E. Lindberg . 2019. “Short Sleep‐Poor Sleep? A Polysomnographic Study in a Large Population‐Based Sample of Women.” Journal of Sleep Research 28, no. 4: e12812. 10.1111/jsr.12812. 30609172 PMC6849745

[jsr70163-bib-0004] Allen, R. P. , D. L. Picchietti , D. Garcia‐Borreguero , et al. 2014. “Restless Legs Syndrome/Willis–Ekbom Disease Diagnostic Criteria: Updated International Restless Legs Syndrome Study Group (IRLSSG) Consensus Criteria—History, Rationale, Description, and Significance.” Sleep Medicine 15, no. 8: 860–873. 10.1016/j.sleep.2014.03.025. 25023924

[jsr70163-bib-0005] American Academy of Sleep Medicine (AASM) . 2014. International Classification of Sleep Disorders. 3rd ed. American Academy of Sleep Medicine.

[jsr70163-bib-0006] American Academy of Sleep Medicine (AASM) . 2023. International Classification of Sleep Disorders—Third Edition, Text Revision (ICSD‐3‐TR). AASM.

[jsr70163-bib-0007] American Psychiatric Association . 2013. Diagnostic and Statistical Manual of Mental Disorders. 5th ed. American Psychiatric Publishing.

[jsr70163-bib-0008] Barrett, L. F. , K. S. Quigley , and P. Hamilton . 2016. “An Active Inference Theory of Allostasis and Interoception in Depression.” Philosophical Transactions of the Royal Society of London. Series B, Biological Sciences 371, no. 1708: 20160011. 10.1098/rstb.2016.0011. 28080969 PMC5062100

[jsr70163-bib-0009] Bastien, C. H. , T. Ceklic , P. St‐Hilaire , et al. 2014. “Insomnia and Sleep Misperception.” Pathologie Biologie 62, no. 5: 241–251. 10.1016/j.patbio.2014.07.003. 25179115

[jsr70163-bib-0010] Bazil, C. W. , B. Legros , and E. Kenny . 2003. “Sleep Structure in Patients With Psychogenic Nonepileptic Seizures.” Epilepsy & Behavior 4, no. 4: 395–398. 10.1016/s1525-5050(03)00120-3. 12899859

[jsr70163-bib-0011] Beck, A. T. , C. H. Ward , M. Mendelson , J. Mock , and J. Erbaugh . 1961. “An Inventory for Measuring Depression.” Archives of General Psychiatry 4: 561–571. 13688369 10.1001/archpsyc.1961.01710120031004

[jsr70163-bib-0012] Berry, R. B. , R. Brooks , C. E. Gamaldo , et al. 2015. The AASM Manual for the Scoring of Sleep and Associated Events: Rules, Terminology and Technical Specifications, Version 2.2. American Academy of Sleep Medicine.

[jsr70163-bib-0013] Brooks, P. L. , and J. Peever . 2016. “A Temporally Controlled Inhibitory Drive Coordinates Twitch Movements During REM Sleep.” Current Biology 26, no. 9: 1177–1182. 10.1016/j.cub.2016.03.013. 27040781

[jsr70163-bib-0014] Butler, M. , O. Shipston‐Sharman , M. Seynaeve , et al. 2021. “International Online Survey of 1048 Individuals With Functional Neurological Disorder.” European Journal of Neurology 28, no. 11: 3591–3602. 10.1111/ene.15018. 34245646

[jsr70163-bib-0015] Buysse, D. J. , C. F. Reynolds 3rd , T. H. Monk , S. R. Berman , and D. J. Kupfer . 1989. “The Pittsburgh Sleep Quality Index: A New Instrument for Psychiatric Practice and Research.” Psychiatry Research 28, no. 2: 193–213. 10.1016/0165-1781(89)90047-4. 2748771

[jsr70163-bib-0016] Cabreira, V. , L. Frostholm , L. McWhirter , J. Stone , and A. Carson . 2023. “Clinical Signs in Functional Cognitive Disorders: A Systematic Review and Diagnostic Meta‐Analysis.” Journal of Psychosomatic Research 173: 111447. 10.1016/j.jpsychores.2023.111447. 37567095

[jsr70163-bib-0017] Carson, A. J. , J. Stone , C. H. Hansen , et al. 2015. “Somatic Symptom Count Scores Do Not Identify Patients With Symptoms Unexplained by Disease: A Prospective Cohort Study of Neurology Outpatients.” Journal of Neurology, Neurosurgery and Psychiatry 86, no. 3: 295–301. 10.1136/jnnp-2014-308234. 24935983

[jsr70163-bib-0018] Corfield, E. C. , N. G. Martin , and D. R. Nyholt . 2016. “Co‐Occurrence and Symptomatology of Fatigue and Depression.” Comprehensive Psychiatry 71: 1–10. 10.1016/j.comppsych.2016.08.004. 27567301

[jsr70163-bib-0019] Cox, R. C. , and B. O. Olatunji . 2020. “Sleep in the Anxiety‐Related Disorders: A Meta‐Analysis of Subjective and Objective Research.” Sleep Medicine Reviews 51: 101282. 10.1016/j.smrv.2020.101282. 32109832

[jsr70163-bib-0020] Demartini, B. , P. Petrochilos , L. Ricciardi , G. Price , M. J. Edwards , and E. Joyce . 2014. “The Role of Alexithymia in the Development of Functional Motor Symptoms (Conversion Disorder).” Journal of Neurology, Neurosurgery, and Psychiatry 85, no. 10: 1132–1137. 10.1136/jnnp-2013-307203. 24610939 PMC4173967

[jsr70163-bib-0021] Demartini, B. , L. Ricciardi , L. Crucianelli , A. Fotopoulou , and M. J. Edwards . 2016. “Sense of Body Ownership in Patients Affected by Functional Motor Symptoms (Conversion Disorder).” Consciousness and Cognition 39: 70–76. 10.1016/j.concog.2015.11.005. 26704189

[jsr70163-bib-0022] Drane, D. L. , N. Fani , M. Hallett , S. S. Khalsa , D. L. Perez , and N. A. Roberts . 2021. “A Framework for Understanding the Pathophysiology of Functional Neurological Disorder.” CNS Spectrums 26: 555–561. 10.1017/S1092852920001789. PMC7930164 32883381

[jsr70163-bib-0023] Edwards, M. J. , R. A. Adams , H. Brown , I. Parees , and K. J. Friston . 2012. “A Bayesian Account of ‘Hysteria’.” Brain 135, no. Pt 11: 3495–3512. 10.1093/brain/aws129. 22641838 PMC3501967

[jsr70163-bib-0024] Finan, P. H. , B. R. Goodin , and M. T. Smith . 2013. “The Association of Sleep and Pain: An Update and a Path Forward.” Journal of Pain 14, no. 12: 1539–1552. 10.1016/j.jpain.2013.08.007. 24290442 PMC4046588

[jsr70163-bib-0025] Forejtova, Z. , T. Serranova , T. Sieger , et al. 2023. “The Complex Syndrome of Functional Neurological Disorder.” Psychological Medicine 53, no. 7: 3157–3167. 10.1017/S0033291721005225. 34991744

[jsr70163-bib-0026] Gelauff, J. M. , J. G. M. Rosmalen , J. Gardien , J. Stone , and M. A. J. Tijssen . 2020. “Shared Demographics and Comorbidities in Different Functional Motor Disorders.” Parkinsonism & Related Disorders 70: 1–6. 10.1016/j.parkreldis.2019.11.018. 31785442

[jsr70163-bib-0027] Graham, C. D. , and S. D. Kyle . 2017. “A Preliminary Investigation of Sleep Quality in Functional Neurological Disorders: Poor Sleep Appears Common, and Is Associated With Functional Impairment.” Journal of the Neurological Sciences 378: 163–166. 10.1016/j.jns.2017.05.021. 28566156

[jsr70163-bib-0028] Gupta, A. , and A. E. Lang . 2009. “Psychogenic Movement Disorders.” Current Opinion in Neurology 22, no. 4: 430–436. 10.1097/WCO.0b013e32832dc169. 19542886

[jsr70163-bib-0029] Hallett, M. , S. Aybek , B. A. Dworetzky , L. McWhirter , J. P. Staab , and J. Stone . 2022. “Functional Neurological Disorder: New Subtypes and Shared Mechanisms.” Lancet Neurology 21, no. 6: 537–550. 10.1016/S1474-4422(21)00422-1. 35430029 PMC9107510

[jsr70163-bib-0030] Hell, D. 2015. “Fatigue and Depression.” Revue Médicale Suisse 11, no. 471: 918–923. 26072598

[jsr70163-bib-0031] Johns, M. W. 1991. “A New Method for Measuring Daytime Sleepiness: The Epworth Sleepiness Scale.” Sleep 14, no. 6: 540–545. 1798888 10.1093/sleep/14.6.540

[jsr70163-bib-0032] Jungilligens, J. , S. Paredes‐Echeverri , S. Popkirov , L. F. Barrett , and D. L. Perez . 2022. “A New Science of Emotion: Implications for Functional Neurological Disorder.” Brain 145, no. 8: 2648–2663. 10.1093/brain/awac204. 35653495 PMC9905015

[jsr70163-bib-0033] Jungilligens, J. , and D. L. Perez . 2024. “Predictive Processing and the Pathophysiology of Functional Neurological Disorder.” In Current Topics in Behavioral Neurosciences, 1–27. Springer Berlin Heidelberg. 10.1007/7854_2024_473. 38755514

[jsr70163-bib-0034] Kannan, S. , A. Dutta , and A. Das . 2025. “Sleep Disorders in Functional Neurological Disorder—A Systematic Review and Meta‐Analysis.” Neurological Sciences 46, no. 4: 1573–1580. 10.1007/s10072-024-07931-9. 39739275 PMC11920331

[jsr70163-bib-0035] Kaplan, K. A. , P. R. Hardas , S. Redline , J. M. Zeitzer , and Sleep Heart Health Study Research Group . 2017. “Correlates of Sleep Quality in Midlife and Beyond: A Machine Learning Analysis.” Sleep Medicine 34: 162–167. 10.1016/j.sleep.2017.03.004. 28522086 PMC5456454

[jsr70163-bib-0036] Keklund, G. , and T. Akerstedt . 1997. “Objective Components of Individual Differences in Subjective Sleep Quality.” Journal of Sleep Research 6, no. 4: 217–220. 10.1111/j.1365-2869.1997.00217.x. 9493520

[jsr70163-bib-0037] Kimura, M. , M. L. Curzi , and C. P. Romanowsi . 2014. “REM Sleep Alteration and Depression.” Archives Italiennes de Biologie 152, no. 2–3: 111–117. 10.12871/000298292014236. 25828683

[jsr70163-bib-0038] Krystal, A. D. 2020. “Sleep Therapeutics and Neuropsychiatric Illness.” Neuropsychopharmacology 45, no. 1: 166–175. 10.1038/s41386-019-0474-9. 31376815 PMC6879486

[jsr70163-bib-0039] Latreille, V. , G. Baslet , R. Sarkis , M. Pavlova , and B. A. Dworetzky . 2018. “Sleep in Psychogenic Nonepileptic Seizures: Time to Raise a Red Flag.” Epilepsy & Behavior 86: 6–8. 10.1016/j.yebeh.2018.07.001. 30032094

[jsr70163-bib-0040] Lim, D. C. , D. R. Mazzotti , K. Sutherland , et al. 2020. “Reinventing Polysomnography in the Age of Precision Medicine.” Sleep Medicine Reviews 52: 101313. 10.1016/j.smrv.2020.101313. 32289733 PMC7351609

[jsr70163-bib-0041] Lopez, R. , L. Barateau , E. Evangelista , and Y. Dauvilliers . 2017. “Depression and Hypersomnia: A Complex Association.” Sleep Medicine Clinics 12, no. 3: 395–405. 10.1016/j.jsmc.2017.03.016. 28778237

[jsr70163-bib-0042] McCarter, S. J. , P. T. Hagen , E. K. St Louis , et al. 2022. “Physiological Markers of Sleep Quality: A Scoping Review.” Sleep Medicine Reviews 64: 101657. 10.1016/j.smrv.2022.101657. 35753151

[jsr70163-bib-0043] Nepozitek, J. , S. Dostalova , G. Vechetova , et al. 2024. “Sleepiness and Comorbid Sleep Disorders in Functional Motor Disorders: A Comparative Study With Central Hypersomnia.” Journal of Sleep Research 33, no. 3: e14098. 10.1111/jsr.14098. 37967854

[jsr70163-bib-0044] Nicholson, T. R. , A. Carson , M. J. Edwards , et al. 2020. “Outcome Measures for Functional Neurological Disorder: A Review of the Theoretical Complexities.” Journal of Neuropsychiatry and Clinical Neurosciences 32, no. 1: 33–42. 10.1176/appi.neuropsych.19060128. 31865871

[jsr70163-bib-0045] Nielsen, G. , L. Ricciardi , A. M. Meppelink , K. Holt , T. Teodoro , and M. Edwards . 2017. “A Simplified Version of the Psychogenic Movement Disorders Rating Scale: The Simplified Functional Movement Disorders Rating Scale (S‐FMDRS).” Movement Disorders Clinical Practice 4, no. 5: 710–716. 10.1002/mdc3.12475. 30363505 PMC6174502

[jsr70163-bib-0046] Pandi‐Perumal, S. R. , J. M. Monti , D. Burman , et al. 2020. “Clarifying the Role of Sleep in Depression: A Narrative Review.” Psychiatry Research 291: 113239. 10.1016/j.psychres.2020.113239. 32593854

[jsr70163-bib-0047] Peever, J. , and P. M. Fuller . 2017. “The Biology of REM Sleep.” Current Biology 27, no. 22: R1237–R1248. 10.1016/j.cub.2017.10.026. 29161567

[jsr70163-bib-0048] Pick, S. , L. H. Goldstein , D. L. Perez , and T. R. Nicholson . 2019. “Emotional Processing in Functional Neurological Disorder: A Review, Biopsychosocial Model and Research Agenda.” Journal of Neurology, Neurosurgery and Psychiatry 90, no. 6: 704–711. 10.1136/jnnp-2018-319201. 30455406 PMC6525039

[jsr70163-bib-0049] Popkirov, S. , J. Stone , and C. P. Derry . 2019. “Abnormal Sleep in Patients With Epileptic or Dissociative (Non‐Epileptic) Seizures: A Polysomnography Study.” European Journal of Neurology 26, no. 2: 255–260. 10.1111/ene.13798. 30168895

[jsr70163-bib-0050] Rakel, R. E. 1999. “Depression.” Primary Care 26, no. 2: 211–224. 10.1016/s0095-4543(08)70003-4. 10318745

[jsr70163-bib-0051] Ricciardi, L. , B. Demartini , L. Crucianelli , C. Krahe , M. J. Edwards , and A. Fotopoulou . 2016. “Interoceptive Awareness in Patients With Functional Neurological Symptoms.” Biological Psychology 113: 68–74. 10.1016/j.biopsycho.2015.10.009. 26528552

[jsr70163-bib-0052] Ricciardi, L. , V. Nistico , E. Andrenelli , et al. 2021. “Exploring Three Levels of Interoception in People With Functional Motor Disorders.” Parkinsonism & Related Disorders 86: 15–18. 10.1016/j.parkreldis.2021.03.029. 33819899

[jsr70163-bib-0053] Riemann, D. , L. B. Krone , K. Wulff , and C. Nissen . 2020. “Sleep, Insomnia, and Depression.” Neuropsychopharmacology 45, no. 1: 74–89. 10.1038/s41386-019-0411-y. 31071719 PMC6879516

[jsr70163-bib-0054] Senaratna, C. V. , J. L. Perret , C. J. Lodge , et al. 2017. “Prevalence of Obstructive Sleep Apnea in the General Population: A Systematic Review.” Sleep Medicine Reviews 34: 70–81. 10.1016/j.smrv.2016.07.002. 27568340

[jsr70163-bib-0055] Serranova, T. , M. Slovak , D. Kemlink , K. Sonka , M. Hallett , and E. Ruzicka . 2019. “Prevalence of Restless Legs Syndrome in Functional Movement Disorders: A Case‐Control Study From The Czech Republic.” BMJ Open 9, no. 1: e024236. 10.1136/bmjopen-2018-024236. PMC6347872 30670516

[jsr70163-bib-0056] Shaffer, C. , C. Westlin , K. S. Quigley , S. Whitfield‐Gabrieli , and L. F. Barrett . 2022. “Allostasis, Action, and Affect in Depression: Insights From the Theory of Constructed Emotion.” Annual Review of Clinical Psychology 18: 553–580. 10.1146/annurev-clinpsy-081219-115627. PMC9247744 35534123

[jsr70163-bib-0057] Slovak, M. , J. Anyz , J. Erlebach , et al. 2022. “Emotional Arousal in Patients With Functional Movement Disorders: A Pupillometry Study.” Journal of Psychosomatic Research 162: 111043. 10.1016/j.jpsychores.2022.111043. 36166959

[jsr70163-bib-0058] Sojka, P. , M. Bares , T. Kasparek , and M. Svetlak . 2018. “Processing of Emotion in Functional Neurological Disorder.” Frontiers in Psychiatry 9: 479. 10.3389/fpsyt.2018.00479. 30344497 PMC6182079

[jsr70163-bib-0059] Sojka, P. , I. Diez , M. Bares , and D. L. Perez . 2021. “Individual Differences in Interoceptive Accuracy and Prediction Error in Motor Functional Neurological Disorders: A DTI Study.” Human Brain Mapping 42, no. 5: 1434–1445. 10.1002/hbm.25304. 33615622 PMC7927304

[jsr70163-bib-0060] Spielberger, C. D. , R. L. Gorsuch , and R. D. Lushene . 1970. STAI: Manual for the State‐Trait Anxiety Inventory. Consulting Psychologists Press.

[jsr70163-bib-0061] Stone, J. , C. Warlow , and M. Sharpe . 2010. “The Symptom of Functional Weakness: A Controlled Study of 107 Patients.” Brain 133, no. Pt 5: 1537–1551. 10.1093/brain/awq068. 20395262

[jsr70163-bib-0062] Sundararajan, T. , G. E. Tesar , and X. F. Jimenez . 2016. “Biomarkers in the Diagnosis and Study of Psychogenic Nonepileptic Seizures: A Systematic Review.” Seizure 35: 11–22. 10.1016/j.seizure.2015.12.011. 26774202

[jsr70163-bib-0063] Teodoro, T. , M. J. Edwards , and J. D. Isaacs . 2018. “A Unifying Theory for Cognitive Abnormalities in Functional Neurological Disorders, Fibromyalgia and Chronic Fatigue Syndrome: Systematic Review.” Journal of Neurology, Neurosurgery and Psychiatry 89, no. 12: 1308–1319. 10.1136/jnnp-2017-317823. 29735513 PMC6288708

[jsr70163-bib-0064] Van den Bergh, O. , M. Witthoft , S. Petersen , and R. J. Brown . 2017. “Symptoms and the Body: Taking the Inferential Leap.” Neuroscience and Biobehavioral Reviews 74, no. Pt A: 185–203. 10.1016/j.neubiorev.2017.01.015. 28108416

[jsr70163-bib-0065] Vanek, J. , J. Prasko , M. Ociskova , et al. 2021. “Sleep Disturbances in Patients With Nonepileptic Seizures.” Nature and Science of Sleep 13: 209–218. 10.2147/NSS.S289190. PMC7896787 33623462

[jsr70163-bib-0066] Vechetova, G. , M. Slovak , D. Kemlink , et al. 2018. “The Impact of Non‐Motor Symptoms on the Health‐Related Quality of Life in Patients With Functional Movement Disorders.” Journal of Psychosomatic Research 115: 32–37. 10.1016/j.jpsychores.2018.10.001. 30470314

[jsr70163-bib-0067] Wichniak, A. , A. Wierzbicka , M. Walecka , and W. Jernajczyk . 2017. “Effects of Antidepressants on Sleep.” Current Psychiatry Reports 19, no. 9: 63. 10.1007/s11920-017-0816-4. 28791566 PMC5548844

[jsr70163-bib-0068] Wu, Y. L. , L. Y. Chang , H. C. Lee , S. C. Fang , and P. S. Tsai . 2017. “Sleep Disturbances in Fibromyalgia: A Meta‐Analysis of Case‐Control Studies.” Journal of Psychosomatic Research 96: 89–97. 10.1016/j.jpsychores.2017.03.011. 28545798

[jsr70163-bib-0069] Yasugaki, S. , H. Okamura , A. Kaneko , and Y. Hayashi . 2025. “Bidirectional Relationship Between Sleep and Depression.” Neuroscience Research 211: 57–64. 10.1016/j.neures.2023.04.006. 37116584

