# Supplementary material for: Sleep in Functional Motor Disorders: A Case–Control Polysomnographic Study
Source: J Sleep Res. 2025 Jul 31;35(2):e70163. doi: 10.1111/jsr.70163 (PMC13003293; doi:10.1111/jsr.70163)
Supplement: Supplementary file 1 — Table S1: Overview of the clinical parameters among the FMD group. Table S2: Comparison of demographic, clinical and polysomnographic parameters in FMD patients with and without insomnia. Table S3: ANCOVA analyses showing a comparison of polysomnographic parameters with BDI‐II, STAI X2 and antidepressant use as covariates. Table S4: ANCOVA with combined influence of covariates—BDI‐II, STAI X2, REM‐reducing and REM‐indifferent antidepressants. [file JSR-35-e70163-s001.docx]

**Supplementary material**

**Supplementary table 1: Overview of the clinical parameters among the FMD group.**

|  | **Medication** | **Disease duration**  **[years]** | **Phenotype** | **Sleep comorbidity** |
| --- | --- | --- | --- | --- |
| Patient 1 | pregabalin, escitalopram | 3 | D, W | PLMS, RLS, insomnia |
| Patient 2 | gabapentin | 7 | G, T, S | severe OSA |
| Patient 3 | pregabalin, risperidone | 1 | W, G, S | mild OSA, RLS, insomnia |
| Patient 4 |  | 3 | T, G |  |
| Patient 5 |  | 6 | D, T, G |  |
| Patient 6 | zolpidem | 6 | T, G, W, S | moderate OSA, RLS, insomnia |
| Patient 7 | paroxetine, topiramate | 3 | G, W, S |  |
| Patient 8 |  | 8 | G, W | severe OSA |
| Patient 9 | mirtazapine | 3 | W, G |  |
| Patient 10 | trazodone | 5 | D, T, G | RLS, insomnia |
| Patient 11 | sertraline, gabapentin | 6 | W, G | mild OSA |
| Patient 12 |  | 4 | D, G, W |  |
| Patient 13 | sertraline | 3 | T, D | moderate OSA |
| Patient 14 | topiramate | 4 | W, T, G | mild OSA, RLS, insomnia |
| Patient 15 |  | 3 | T, G, W | insomnia |
| Patient 16 |  | 3 | G, W | RLS |
| Patient 17 |  | 1 | G, W | insomnia |
| Patient 18 |  | 8 | T, G, W | insomnia |
| Patient 19 |  | 1 | T, G, D, W, S | insomnia |
| Patient 20 | citalopram | 8 | W, G | mild OSA, RLS, insomnia |
| Patient 21 |  | 1 | T | moderate OSA, PLMS, insomnia |
| Patient 22 |  | 11 | M, G, W |  |
| Patient 23 | trazodone, tramadol | 5 | G, W | moderate OSA, RLS, insomnia |
| Patient 24 |  | 3 | G, T, W |  |
| Patient 25 | sertraline | 3 | D, T, G | severe OSA, insomnia |
| Patient 26 |  | 3 | G, D, W, M | RLS, insomnia |
| Patient 27 | pregabalin, trazodone | 1 | G, T, W | severe OSA, RLS, insomnia |
| Patient 28 | venlafaxine | 1 | W, T, G | severe OSA, PLMS, insomnia |
| Patient 29 |  | 6 | G, T, D, W | severe OSA |
| Patient 30 |  | 1 | W, T, G |  |
| Patient 31 |  | 12 | T, G, W, S | insomnia |
| Patient 32 |  | 2 | G, W |  |
| Patient 33 | venlafaxine, mirtazapine, tramadol | 1 | G, T, D, W | insomnia |
| Patient 34 | trazodone, escitalopram | 4 | D, T, G, W | moderate OSA, insomnia |
| Patient 35 |  | 6 | D, T, G, W |  |
| Patient 36 | mirtazapine, hydroxyzine | 4 | W, G | insomnia |
| Patient 37 | escitalopram, mirtazapine | 4 | G, T, D, W | moderate OSA, insomnia |

The clinical manifestation of FMD is stated in the phenotype column. The dominant sign is the first one; the following signs are additional. Only drugs potentially affecting sleep are noted in the medication column.

Legend: T – tremor; G – gait disorder; D – dystonia; W – weakness; M – myoclonus; S – speech disturbance; OSA – obstructive sleep apnea; PLMS – periodic limb movements syndrome; RLS – restless legs syndrome.

**Supplementary table 2: Comparison of demographic, clinical, and polysomnographic parameters in FMD patients with and without insomnia.**

|  | Insomnia + | | Insomnia - | |  |  |
| --- | --- | --- | --- | --- | --- | --- |
|  | Mean | SD | Mean | SD | p | p-adj. |
| Age | 46.4 | 10.9 | 50.4 | 9.9 | 0.373 | 0.843 |
| Gender (F/M) | 14/7 | | 13/3 | | 0.461 | 0.843 |
| BMI | 26.9 | 4.6 | 27.9 | 3.9 | 0.540 | 0.843 |
| S-FMDRS | 13.7 | 7.3 | 15.9 | 8.5 | 0.549 | 0.843 |
| **Questionnaires** |  |  |  |  |  |  |
| PSQI | 25.7 | 5.3 | 24.2 | 8.4 | 0.936 | 0.936 |
| ESS | 9.3 | 5.3 | 11.8 | 5.9 | 0.227 | 0.843 |
| BDI-II | 18.6 | 9.7 | 18.2 | 13.8 | 0.724 | 0.895 |
| STAI X2 | 50.7 | 9.1 | 47.8 | 9.9 | 0.269 | 0.843 |
| **Polysomnography** |  |  |  |  |  |  |
| Total sleep time | 346.7 | 88.9 | 331.8 | 80.5 | 0.387 | 0.843 |
| Sleep latency | 23.6 | 17.7 | 29.8 | 36.1 | 0.783 | 0.895 |
| Sleep efficiency | 71.7 | 16.7 | 69.0 | 17.3 | 0.592 | 0.843 |
| Sleep stage R latency | 156.4 | 79.1 | 174.0 | 94.9 | 0.498 | 0.843 |
| AHI | 15.1 | 15.2 | 14.9 | 19.2 | 0.592 | 0.895 |
| ODI | 8.6 | 7.7 | 10.7 | 15.3 | 0.854 | 0.895 |
| Wake ratio (%) | 25.3 | 16.9 | 26.9 | 15.7 | 0.751 | 0.895 |
| Sleep stage N1 ratio (%) | 7.3 | 4.1 | 5.9 | 1.6 | 0.509 | 0.843 |
| Sleep stage N2 ratio (%) | 34.4 | 10.6 | 33.2 | 9.2 | 0.613 | 0.843 |
| Sleep stage N3 ratio (%) | 19.3 | 10.7 | 20.4 | 8.7 | 0.294 | 0.843 |
| Sleep stage R ratio (%) | 13.8 | 7.6 | 13.6 | 6.4 | 0.854 | 0.895 |
| RLS (+/-) | 11/10 | | 1/15 | | **0.004** | 0.088 |
| PLMI | 4.6 | 8.3 | 1.8 | 3.8 | 0.255 | 0.843 |
| Arousal index | 25.9 | 12.5 | 19 | 10.1 | 0.147 | 0.843 |

Data presented as mean and standard deviation, except for gender and RLS, where absolute counts are provided.

Legend: F – females; M – males; BMI – body mass index; S-FMDRS - Simplified Functional Movement Disorders Rating Scale PSQI - Pittsburgh sleep quality index; ESS – Epworth sleepiness scale; BDI-II – Beck depression inventory, second edition; STAI X2 – State-Trait Anxiety Inventory; AHI – apnea-hypopnea index; ODI – oxygen desaturation index; N1, 2, 3 – non-rapid eye movement sleep stage 1, 2, 3, R – rapid eye movement sleep; RLS – restless legs syndrome; PLMI – periodic limb movements index; FMD – functional motor disorder; SD – standard deviation; adj. – adjusted; p-values < 0.05 are **bolded**; none remained significant after Benjamini-Hochberg false discovery rate adjustment.

**Supplementary table 3: ANCOVA analyses showing a comparison of polysomnographic parameters with BDI-II, STAI X2, and antidepressant use as covariates.**

|  | BDI-II | | | STAI X2 | | | R-reducing AD | | | | R-indifferent AD | | |
| --- | --- | --- | --- | --- | --- | --- | --- | --- | --- | --- | --- | --- | --- |
|  | F | p | Partial Eta Squared | F | p | Partial Eta Squared | F | p | Partial Eta Squared | F | | p | Partial Eta Squared |
| Total sleep time | 4.43 | **0.039** | 0.059 | 1.68 | 0.199 | 0.023 | 2.06 | 0.156 | 0.028 | 2.04 | | 0.158 | 0.028 |
| Sleep latency | 8.80 | **0.004** | 0.110 | 4.40 | **0.040** | 0.058 | 4.57 | **0.036** | 0.060 | 4.05 | | **0.048** | 0.054 |
| Sleep efficiency | 7.16 | **0.009** | 0.092 | 3.85 | 0.054 | 0.051 | 5.57 | **0.021** | 0.073 | 5.64 | | **0.020** | 0.074 |
| Sleep stage R latency | 5.63 | **0.020** | 0.074 | 6.43 | **0.013** | 0.084 | 9.42 | **0.003** | 0.119 | 9.42 | | **0.003** | 0.119 |
| AHI | 0.36 | 0.549 | 0.005 | 0.15 | 0.697 | 0.002 | 0.15 | 0.697 | 0.002 | 1.29 | | 0.260 | 0.018 |
| ODI | 0.33 | 0.567 | 0.005 | 0.11 | 0.744 | 0.002 | 0.97 | 0.327 | 0.014 | 1.68 | | 0.199 | 0.023 |
| Wake ratio (%) | 5.90 | **0.018** | 0.077 | 3.20 | 0.078 | 0.043 | 5.36 | **0.024** | 0.070 | 5.30 | | **0.024** | 0.070 |
| Sleep stage N1 ratio (%) | 3.10 | 0.082 | 0.042 | 2.08 | 0.154 | 0.028 | 2.35 | 0.130 | 0.032 | 0.97 | | 0.327 | 0.014 |
| Sleep stage N2 ratio (%) | 2.19 | 0.144 | 0.030 | 2.48 | 0.120 | 0.034 | 3.01 | 0.087 | 0.041 | 1.18 | | 0.281 | 0.016 |
| Sleep stage N3 ratio (%) | 0.07 | 0.799 | 0.001 | 0.79 | 0.378 | 0.011 | 0.003 | 0.956 | 0.000 | 0.90 | | 0.347 | 0.012 |
| Sleep stage R ratio (%) | 4.20 | **0.044** | 0.056 | 5.32 | **0.024** | 0.070 | 4.01 | **0.049** | 0.053 | 3.25 | | 0.076 | 0.044 |
| PLMI | 4.52 | **0.037** | 0.060 | 1.83 | 0.181 | 0.025 | 0.39 | 0.537 | 0.005 | 2.22 | | 0.140 | 0.030 |
| Arousal index | 2.10 | 0.153 | 0.036 | 0.24 | 0.624 | 0.004 | 1.14 | 0.290 | 0.020 | 1.92 | | 0.171 | 0.033 |

Legend: BDI-II – Beck depression inventory, second edition; STAI X2 – State-Trait Anxiety Inventory; AD – antidepressant drugs; AHI – apnea-hypopnea index; ODI – oxygen desaturation index; N1, 2, 3 – non-rapid eye movement sleep stage 1, 2, 3, R – rapid eye movement sleep; PLMI – periodic limb movements index; p-values < 0.05 are **bolded**.

**Supplementary table 4: ANCOVA with combined influence of covariates – BDI-II, STAI X2, REM-reducing, and REM-indiferent antidepressants**

|  | F | p | Partial Eta Squared |
| --- | --- | --- | --- |
| Total sleep time | 2.716 | 0.104 | 0.38 |
| Sleep latency | 6.683 | **0.012** | 0.089 |
| Sleep efficiency | 4.947 | **0.029** | 0.068 |
| Sleep stage R latency | 4.887 | **0.030** | 0.068 |
| AHI | 0.364 | 0.548 | 0.005 |
| ODI | 0.34 | 0.562 | 0.005 |
| Wake ratio (%) | 3.942 | 0.051 | 0.055 |
| Sleep stage N1 ratio (%) | 2.811 | 0.098 | 0.04 |
| Sleep stage N2 ratio (%) | 1.633 | 0.206 | 0.023 |
| Sleep stage N3 ratio (%) | 0.028 | 0.868 | 0 |
| Sleep stage R ratio (%) | 4.253 | **0.043** | 0.059 |
| PLMI | 4.199 | **0.044** | 0.058 |
| Arousal index | 0.78 | 0.381 | 0.015 |

Legend: AHI – apnea-hypopnea index; ODI – oxygen desaturation index; N1, 2, 3 – non-rapid eye movement sleep stage 1, 2, 3, R – rapid eye movement sleep; PLMI – periodic limb movements index; p-values < 0.05 are **bolded**.
